# Association between systemic inflammation indicators and psoriasis: a cross-sectional study from NHANES

**DOI:** 10.3389/fimmu.2025.1556487

**Published:** 2025-03-13

**Authors:** Huizi Xiong, Zengyang Yu

**Affiliations:** 1 Department of Dermatology, The Fourth Affiliated Hospital of Soochow University, Suzhou Medical College, Soochow University, Suzhou, China; 2 Department of Dermatology, Shanghai Tenth People’s Hospital, Tongji University School of Medicine, Shanghai, China; 3 Institute of Psoriasis, Tongji University School of Medicine, Shanghai, China

**Keywords:** NHANES, psoriasis, NPAR, NHR, NLR

## Abstract

**Objectives:**

To Investigate the association between systemic inflammatory indicators and psoriasis in the adult population of the United States.

**Methods:**

We analyzed data from 16,575 adults in the National Health and Nutrition Examination Survey (NHANES) from 2003–2006 and 2009–2014. Six inflammatory ratios—neutrophil percentageto-albumin ratio (NPAR), neutrophil-to-lymphocyte ratio (NLR), neutrophil-to-high-density lipoprotein cholesterol ratio (NHR), lymphocyte-to-high-density lipoprotein cholesterol ratio (LHR), platelet-to-high-density lipoprotein cholesterol ratio (PHR), and monocyte-to-high-density lipoprotein cholesterol ratio (MHR). Furthermore, subgroup analyses were performed to investigate whether these results remained true among various demographic groups. Finally, the predictive efficacy of inflammatory indicators was assessed through AUC values and ROC curves.

**Results:**

Among the study participants, 432 (2.6%) had psoriasis. There was a remarkable positive association found between psoriasis and NLR, NHR, and NPAR. After adjusting for various confounding factors, it was found that each 10-unit increase in NPAR was associated with a 90% higher chance of developing psoriasis (OR=1.90, 95% CI 1.11-3.26). Similarly, the odds of psoriasis prevalence increased by 10% for every unit rise in NLR (OR=1.10, 95% CI 1.12-1.18). After full adjustment, however, there was no discernible distinction between psoriasis and NHR (OR=1.03, 95% CI 0.98-1.08). Furthermore, the study identified a nonlinear relationship between psoriasis and systemic inflammation indicators like NPAR, NLR, and NHR, with specific breakpoints at 16.386, 3.269, and 4.286, respectively. Subgroup analysis provided additional evidence that this association remained consistent for different demographic groupings. ROC analysis demonstrated that NLR and NPAR showed better accuracy in predicting psoriasis prevalence.

**Conclusion:**

The study indicates a positive affiliation between NPAR, NLR, and the occurrence of psoriasis. Nevertheless, to confirm these discoveries and investigate the underlying mechanisms, more extensive prospective research is necessary.

## 1 Introduction

Psoriasis is a complex and multifaceted chronic inflammatory cutaneous disorder that affects a significant portion of the global population. It is characteristic scaly patches can have a profound impact on both individuals and society, as it is associated with a range of other health issues, including psoriatic arthritis, cardiovascular problems, metabolic abnormalities, obesity, gastrointestinal disorders such as inflammatory bowel disease, and mental health concerns (1). The immune system plays a crucial role in the development and progression of psoriasis. In particular, Th17 cells and dendritic cells (DCs) are known to be central to this process. These immune cells, along with others such as mast cells, monocytes, neutrophils, innate lymphoid cells (ILCs), and macrophages, are all interconnected in the inflammatory process that drives the disease (2).

Recent investigations highlighted the chronic inflammatory nature of psoriasis with the immune system playing a crucial role in its pathophysiology (3). The distinct leukocyte distribution in the blood of psoriasis patients, characterized by an increase in neutrophil counts (4), is a notable finding that supports this understanding. The recognition of PLR (platelet-to-lymphocyte ratio) as a rapid and reliable indicator for identifying subclinical inflammatory diseases like systemic lupus erythematosus and heart failure is also significant (5). This suggests that PLR may have potential diagnostic value in the context of psoriasis as well.

The NPAR (neutrophil-to-albumin ratio) is indeed an intriguing indicator that combines the quantities of albumin and neutrophils. It has shown promise as a technique for predicting mortality in various states such as acute myocardial infarction (AMI) (6), cardiogenic shock (7), coronary artery disease (8), and heart failure in the intensive care unit (9). Similarly, other ratios such as neutrophil-percentage-to-albumin ratio (NPAR), the neutrophil-to-high-density lipoprotein cholesterol ratio (NHR), lymphocyte-to-high-density lipoprotein cholesterol ratio (LHR), monocyte-to-high-density lipoprotein cholesterol ratio (MHR), and platelet-to-high-density lipoprotein cholesterol ratio (PHR) have also been identified as potential markers of inflammatory factors that play a critical role in many diseases (10, 11). High-density lipoprotein cholesterol (HDL-C) is known for its anti-inflammatory properties. However, both its levels and function are compromised during inflammatory states. Furthermore, HDLC engages in complex regulatory interactions with various immune cell types.

The recent findings that psoriasis patients have higher levels of CRP (C-reactive protein), MHR (monocyte-to-hemoglobin ratio), NLR (neutrophil-to-lymphocyte ratio), and MLR (monocyte-to-lymphocyte ratio), and that these levels are positively correlated with the severity of the psoriasis condition, are significant (12). Interestingly, CRP, a well-known inflammation biomarker, was identified to be uniquely associated with MHR (13). Although many of the above inflammatory factors have been shown to correlate with psoriasis, there has been no investigation into the relationship between psoriasis and NPAR (neutrophil-to-albumin ratio) or NHR (neutrophil-to-hemoglobin ratio). This study is the first to investigate the correlation analysis of NPAR, NHR and other related inflammatory factors with the incidence of psoriasis, providing a new basis for predicting the development of psoriasis.

## 2 Methods

### 2.1 Population under investigation

**Figure 1:**
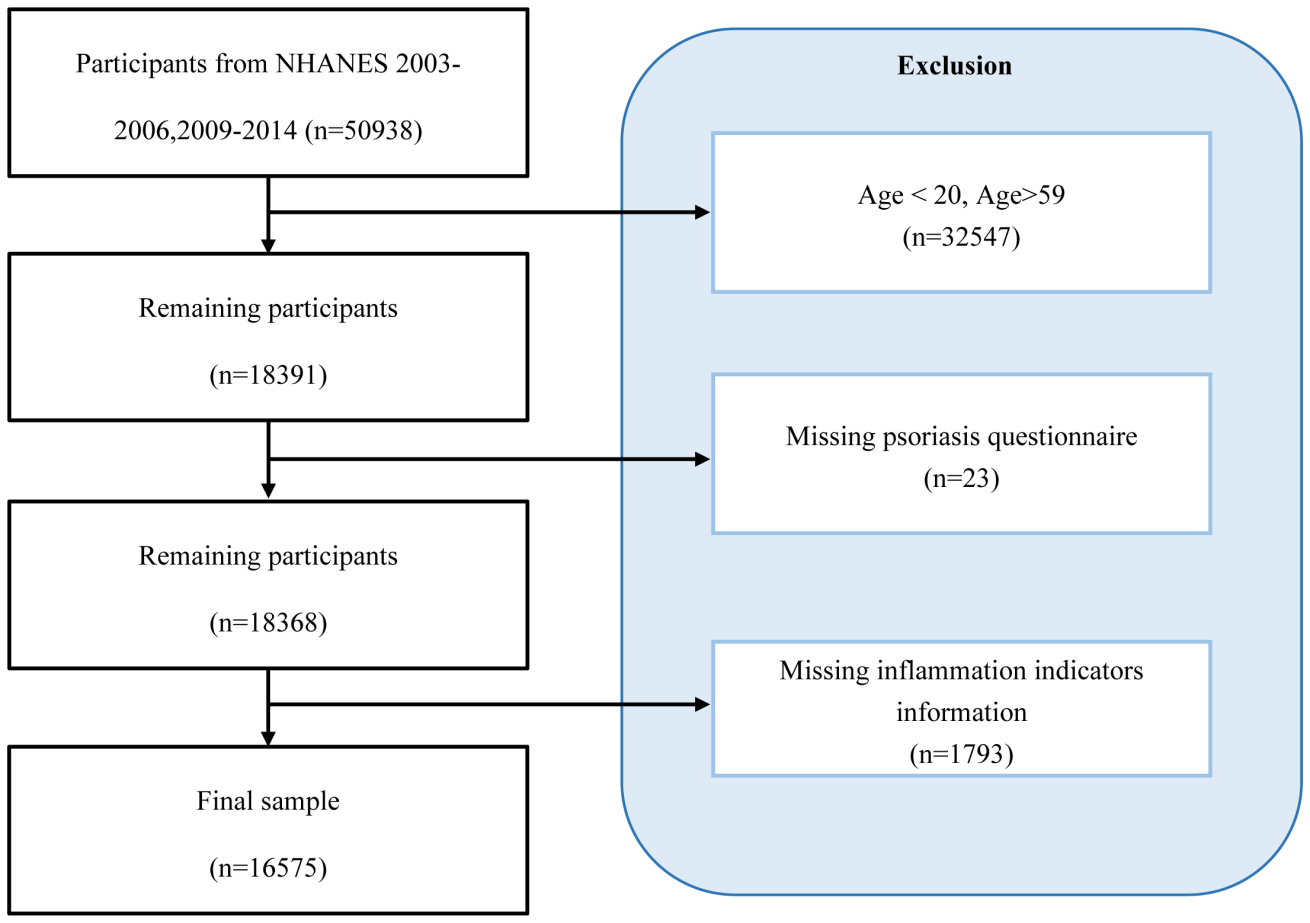
Participant selection flowchart for this study.

The survey in question gathered comprehensive information on demographic data, economic conditions, nutrition, and general health, creating a valuable dataset for studying public health trends and addressing emerging health issues. For this study, we used publicly available data from five two-year NHANES cycles (spanning from 2003-2006 and 2009-2014), focusing on adults aged 20 to 59. A total of 16,575 individuals participated in both the “complete blood count, high-density lipoprotein cholesterol, albumin” examination and the “Psoriasis” questionnaire. Participants were excluded if they lacked specific psoriasis assessment data, had insufficient information to calculate inflammation indicators NPAR, NLR, NHR, LHR, PHR, and MHR, or were outside the specified age range. Ethical clearance for the NHANES project was provided by the National Center for Health Statistics, and all participants gave written consent. In the final analysis, the research group consisted of 432 participants with psoriasis and 16,143 without, making up a total sample size of 16,575 adults (**Figure 1**).

### 2.2 Measurement of systemic inflammation indicators NPAR, NHR, LHR, PHR, and MHR

The relevant laboratory measures were obtained from the NHANES database. HDL-C levels were assessed using an automatic device, with venous blood samples collected after an 8-hour fast. The standard reference range for HDL-C is 1.3–1.5 mmol/L for females and 1.0–1.5 mmol/L for males. White blood cell classification is carried out via the Coulter VCS system. The system incorporates automated mixing and dilution techniques for sample preparation, along with a single-beam photometric method forhemoglobin quantification. Additionally, it is employed for cell counting and molecular weight evaluations. The NLR for each individual was calculated by dividing the total absolute neutrophil count by the total absolute lymphocyte count within the WBC (White Blood Cell) population. Identical blood samples and specific formulas were utilized to determine the NPAR, NHR, LHR, PHR, and MHR, either separately or collectively. These ratios were calculated using the following formulas:


$$\text{NPAR}:\;\left(\text{Percent neutrophils of total leukocyte count }\left[\%\right]\right)\times 100/\text{Albumin }\left(\text{g}/\text{dL}\right);\text{NHR}:\text{ Neutrophil counts }(10^{9}/\text{L})/\text{HDL }(\text{mmol}/\text{L});\text{LHR}:\text{ Lymphocyte counts }(10^{9}/\text{L})/\text{HDL }(\text{mmol}/\text{L});\text{ PHR}:\text{ Platelet counts }(10^{9}/\text{L})/\text{HDL }(\text{mmol}/\text{L});\text{ MHR}:\text{ Monocyte counts }(10^{9}/\text{L})/\text{HDL }(\text{mmol}/\text{L});\text{ NLR}:\text{ Neutrophil counts }(10^{9}/\text{L})/\text{Lymphocyte counts }(10^{9}/\text{L})$$

Participants were categorized into four groups based on quartiles: Quantile 1, Quantile 2, Quantile 3, and Quantile 4s. These groups were established according to their respective levels of NPAR, NHR, PHR, MHR, LHR, and NLR.

### 2.3 Psoriasis definition

The diagnosis of psoriasis was contingent upon the responses provided in the “medical conditions” section of the questionnaire. Specifically, participants who responded affirmatively to the inquiry, “Has a doctor ever informed you that you have psoriasis?” were deemed to have psoriasis, provided they confirmed that the diagnosis was issued by a medical expert.

### 2.4 Covariates

In this research, a meticulously curated selection of covariates was assembled. These covariates encompassed a wide array of demographic characteristics, such as age, gender (distinctively categorized as male or female), marital status, educational attainment spanning from less than high school to high school, post-secondary education, and other classifications, racial identity segmented as non-Hispanic White, non-Hispanic Black, Mexican American, or others along with the income-to-poverty ratio (PIR). Furthermore, a diverse range of medical conditions was included, such as hypertension, hyperlipidemia, diabetes, cardiovascular disease (CVD), and cancer. Lifestyle and health-related factors were also integral parts of the analysis, encompassing body mass index (BMI), smoking behavior determined by whether participants had smoked more than 100 cigarettes in their lifetime, and alcohol consumption patterns assessed by whether they had consumed at least 12 alcoholic beverages in the past year. In terms of defining health conditions, diabetes and CVD were diagnosed based on self-reported, physician-confirmed diagnoses. In particular, diabetes was considered present if participants had a prior doctor-diagnosed case, while CVD encompassed heart failure, coronary artery disease (CAD), angina, heart attack, and stroke. Hyperlipidemia was defined using National Cholesterol Education Program criteria, incorporating LDL cholesterol ≥ 4.1 mmol/L, HDL-C ≤ 1.0 mmol/L in men or ≤ 1.3 mmol/L in women, triglycerides ≥ 2.3 mmol/L, total cholesterol ≥ 6.2 mmol/L, or current use of cholesterol-lowering medication. Hypertension was diagnosed if participants had an average systolic blood pressure ≥130 mmHg, diastolic blood pressure ≥80 mmHg, or were taking prescribed medication for high blood pressure. The laboratory data collected was extensive, including measurements for neutrophil count, lymphocyte count, albumin, monocyte count, cholesterol, and triglycerides.

### 2.5 Statistical analysis

Statistical procedures for this study were meticulously adhered to the NHANES guidelines, accounting for sample weights, clustering, and stratification.

Inflammation biomarkers were stratified into four quartiles, and Chi-square and t-tests were used to compare baseline demographic and clinical characteristics across these quartiles.

To assess the association between inflammatory biomarkers and psoriasis, we developed three multivariable logistic regression models. The first model served as a baseline, examining the relationship between biomarkers and psoriasis without adjusting for any covariates. The second model was adjusted for demographic variables, including age, gender, and ethnicity. The third and most comprehensive model further incorporated socioeconomic factors, lifestyle habits, and medical histories, such as PIR, education level, smoking, marital status, alcohol use, and medical conditions such as diabetes, hypertension, hyperlipidemia, cancer, and cardiovascular disease.

A trend analysis across quartiles of inflammatory biomarkers was performed to examine the consistency of the correlations. Additionally, we performed, we performed subgroup assessments to explore potential variations in the relationship between psoriasis and inflammation biomarkers across different demographic, socioeconomic, and clinical strata.

Furthermore, we utilized smoothing curves and generalized additive models (GAMs) to examine potential nonlinear relationships. We employed a comparison between segmented regression models and a single linear model, using the log-likelihood ratio test to detect threshold effects. Breakpoints were identified through a recursive two-step process.

The predictive accuracy of inflammatory markers for psoriasis was evaluated using the area under the receiver operating characteristic curve (AUC-ROC). Statistical significance was determined using a two-sided P-value threshold of < 0.05. All statistical analyses were performed using R (version 4.2) and Empowerstats package (version 5.0), ensuring the robustness and reproducibility of our findings.

## 3 Results

### 3.1 Baseline characteristics

Among the 16,575 participants in our study, 432 (2.61%) had a previous diagnosis of psoriasis. There were 52% females and 48% males, with an average (SD) age of 38.76 ± 11.37. Ethnic breakdown showed that 43% were non-Hispanic White, 21%non-Hispanic Black, 18% Mexican American, and 7.7% other Hispanic.

Regarding inflammatory biomarkers, the mean NPAR score for all individuals was 13.73± 2.60, the NLR averaged 2.16± 1.05, and the NHR stood at 3.51± 1.86 across the entire cohort.

Our analysis further indicated that individuals with a history of psoriasis were notably more inclined towards having a higher education level and higher smoking status. They were also more likely to suffer from comorbidities such as hypertension, hyperlipidemia, and cancer, as indicated by statistically significant p-values (**Table 1**).

**Table 1:** The baseline characteristics of participants enrolled in the NHANES cycles spanning from 2003-2006 and 2009-2014.

Variables	Overall	Psoriasis		*P*-value
	(n=16575)	NO (n=16143)	YES (n=432)	
**Gender, n (%)**				0.037
Male	7,945 (48%)	7,714 (48%)	231 (55%)	
Female	8,630 (52%)	8,429 (52%)	201 (45%)	
**Age, Mean ± SD**	38.76± (11.37)	38.75± (11.38)	39.20± (11.31)	0.495
**Age strata, n (%)**				0.134
20-39	8,626 (52%)	8,418 (52%)	208 (48%)	
40-59	7,949 (48%)	7,725 (48%)	224 (52%)	
**Race, n (%)**				0.613
Mexican American	2,911 (18%)	2,827 (18%)	84 (19%)	
Other Hispanic	1,283 (7.7%)	1,255 (7.8%)	28 (7.2%)	
Non-Hispanic White	7,127 (43%)	6,948 (43%)	179 (40%)	
Non-Hispanic Black	3,524 (21%)	3,429 (21%)	95 (20%)	
Other Race	1,730 (10%)	1,684 (10%)	46 (13%)	
**Education level, n (%)**				0.003
Less than 9th grade	1,294 (8.2%)	1,247 (8.1%)	47 (12%)	
9-11th grade	2,400 (15%)	2,339 (15%)	61 (13%)	
High school graduate	3,732 (23%)	3,650 (23%)	82 (17%)	
Some college or associates degree	5,237 (31%)	5,109 (31%)	128 (28%)	
College graduate or above	3,912 (23%)	3,798 (23%)	114 (30%)	
**Marital status, n (%)**				0.639
Married/Living with a partner	10,126 (61%)	9,855 (61%)	271 (63%)	
Divorced/Separated/Widowed	2,409 (15%)	2,339 (15%)	70 (15%)	
Never married	4,040 (25%)	3,949 (25%)	91 (22%)	
**Income-to-poverty ratio, n (%)**				0.070
≤1.3	5,327 (33%)	5,197 (33%)	130 (31%)	
1.3-3.5	6,086 (37%)	5,933 (37%)	153 (33%)	
>3.5	5,162 (30%)	5,013 (29%)	149 (35%)	
**BMI, n (%)**				0.502
<25	5,197 (35%)	5,079 (35%)	118 (32%)	
25-30	5,342 (34%)	5,199 (34%)	143 (34%)	
≥30	6,036 (31%)	5,865 (31%)	171 (34%)	
**Smoking, n (%)**				<0.001
Yes	7,086 (44%)	6,860 (44%)	226 (54%)	
No	9,489 (56%)	9,283 (56%)	206 (46%)	
**Alcohol use, n (%)**				0.984
Yes	12,351 (79%)	12,030 (79%)	321 (79%)	
No	4,224 (21%)	4,113 (21%)	111 (21%)	
**Hypertension, n (%)**				0.003
Yes	3,683 (22%)	3,550 (22%)	133 (30%)	
No	12,892 (78%)	12,593 (78%)	299 (70%)	
**Hyperlipidemia, n (%)**				<0.001
Yes	4,670 (30%)	4,505 (29%)	165 (40%)	
No	11,905 (70%)	11,638 (71%)	267 (60%)	
**Coronary heart disease, n (%)**				0.514
Yes	187 (1.1%)	176 (1.1%)	11 (1.4%)	
No	16,388 (99%)	15,967 (99%)	421 (99%)	
**Cancer, n (%)**				0.016
Yes	638 (5.0%)	607 (4.9%)	31 (8.3%)	
No	15,937 (95%)	15,536 (95%)	401 (92%)	
**Diabetes, n (%)**				0.594
Yes	1,075 (5.5%)	1,041 (5.5%)	34 (6.2%)	
No	15,500 (94%)	15,102 (94%)	398 (94%)	
**White blood cell (10^3^ cells/μL)**	7.32± (2.20)	7.33± (2.21)	7.11± (1.98)	0.144
**Platelet (10^3^ cells/μL)**	255.92± (65.20)	255.79± (65.13)	260.29± (67.21)	0.310
**Lymphocyte (10^3^ cells/μL)**	2.17± (0.81)	2.17± (0.82)	2.10± (0.66)	0.211
**Monocyte (10^3^ cells/μL)**	0.55± (0.18)	0.54± (0.18)	0.55± (0.16)	0.171
**Neutrophil (10^3^ cells/μL)**	4.37± (1.74)	4.36± (1.74)	4.52± (1.65)	0.059
**Neutrophils percent (%)**	58.52± (8.97)	58.48± (8.97)	59.80± (8.63)	0.004
**Albumin (g/L)**	42.98± (3.50)	42.99± (3.50)	42.75± (3.34)	0.147
**HDL -C (mmol/L)**	1.37± (0.41)	1.37± (0.41)	1.34± (0.41)	0.179
**NPAR (dL/g)**	13.73± (2.60)	13.72± (2.60)	14.11± (2.56)	0.003
**NLR (10^9^/mmol)**	2.16± (1.05)	2.16± (1.05)	2.29± (0.94)	<0.001
**NHR (10^9^/mmol)**	3.51± (1.86)	3.51± (1.86)	3.70± (1.77)	0.014
**LHR (10^9^/mmol)**	1.75± (0.93)	1.75± (0.94)	1.72± (0.82)	0.800
**PHR (10^9^/mmol)**	202.85± (79.16)	202.65± (79.20)	209.33± (77.83)	0.063
**MHR (10^9^/mmol)**	0.44± (0.22)	0.44± (0.22)	0.45± (0.20)	0.098

*p* < 0.05 was deemed statistically significant; NHANES, National Health and Nutrition Examination Survey.

BMI, body mass index; HDL-C, high-density lipoprotein cholesterol; NPAR, neutrophil-percentage-to-albumin ratio; NLR, neutrophil-to-lymphocyte ratio; NHR, neutrophil-to-high-density lipoprotein cholesterol ratio; LHR, lymphocyte-to-high-density lipoprotein cholesterol ratio; PHR, platelet-to-high-density lipoprotein cholesterol ratio; MHR, Monocyte-to-high-density lipoprotein cholesterol ratio.

### 3.2 Psoriasis exhibits a correlation with inflammation indicators

**Figure 2:**
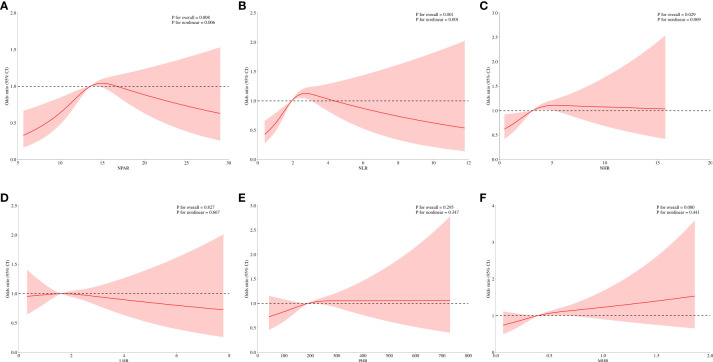
Smooth curve fitting for systemic inflammatory indicators with psoriasis. **(A)** NPAR and psoriasis; **(B)** NLR and psoriasis; **(C)** NHR and psoriasis; **(D)** LHR and psoriasis; **(E)** PHR and psoriasis; **(F)** MHR and psoriasis.

The current analysis assessed the relationship between psoriasis prevalence and systemic inflammatory indicators across three models (**Table 2**). Model 1 reveals a compelling correlation: for every increment of 10 units in NPAR, there is a notable 73% surge in the odds of developing psoriasis (odds ratio [OR] =1.73, with a 95% confidence interval [CI] of 1.10-2.72). This significant association persists even after adjusting for relevant covariates in Models 2 (OR=1.75, 95% CI 1.12-2.74) and 3 (OR=1.90, 95% CI 1.11-3.26). The results from the quartile analysis of NPAR confirmed a consistent significant relationship across all models. Likewise, in Model 1, a 5% higher likelihood of psoriasis was seen with each increase in NHR. While this relationship undergoes slight moderation after covariate adjustment in Model 2 (OR=1.05, 95% CI 1.01-1.10), it remains statistically significant. However, this association was no longer significant in Model 3 for NHR (OR=1.03, 95% CI 0.98-1.08). When examining other systemic inflammatory indicators, such as NLR, LHR, PHR, and MHR, no statistically significant correlations with psoriasis were observed for LHR, PHR, or MHR. The smoothed curve fitting confirmed the nonlinear relationship between NPAR, NLR, NHR, and psoriasis. Notably, the breakpoints for NPAR, NLR, and NHR were found to be 16.386, 3.269, and 4.286, respectively (**Table 3**). Specifically, the risk of psoriasis was positively associated with NPAR levels below 16.386 (OR=1.119, 95% CI 1.062-1.181) and NLR levels below 3.269 (OR=1.415, 95% CI 0.998-1.161) (**Figure 2**).

**Table 2:** Associations between systemic inflammatory indicators and psoriasis: multivariable logistic regression analysis of NHANES 2003–2006 and 2009-2014.

	Model 1 OR^1^ (95% CI^2^)	Model 2 OR^1^ (95% CI^2^)	Model 3 OR^1^ (95% CI^2^)
NPAR			
Continuous	1.06 (1.01, 1.11)	1.06 (1.01, 1.11)	1.07 (1.01, 1.13)
Categories			
Q1	Reference	Reference	Reference
Q2	0.99 (0.69, 1.42)	1.00 (0.70, 1.43)	0.99 (0.69, 1.41)
Q3	1.26 (0.94, 1.69)	1.26 (0.94, 1.70)	1.25 (0.92, 1.70)
Q4	1.53 (1.09, 2.14)	1.54 (1.11, 2.15)	1.56 (1.11, 2.19)
*P* for trend	0.005	0.004	0.006
NPAR/10			
Continuous	1.73 (1.10, 2.72)	1.75 (1.12, 2.74)	1.90 (1.11, 3.26)
Categories			
Q1	Reference	Reference	Reference
Q2	0.99 (0.69, 1.42)	1.00 (0.70, 1.43)	0.99 (0.69, 1.41)
Q3	1.26 (0.94, 1.69)	1.26 (0.94, 1.70)	1.25 (0.92, 1.70)
Q4	1.53 (1.09, 2.14)	1.54 (1.11, 2.15)	1.56 (1.11, 2.19)
*P* for trend	0.005	0.004	0.006
NLR			
Continuous	1.11 (1.03, 1.19)	1.11 (1.04, 1.20)	1.10 (1.02, 1.18)
Categories			
Q1	Reference	Reference	Reference
Q2	0.98 (0.68, 1.42)	0.99 (0.69, 1.43)	0.98 (0.68, 1.41)
Q3	1.24 (0.88, 1.73)	1.24 (0.88, 1.74)	1.22 (0.87, 1.71)
Q4	1.66 (1.19, 2.32)	1.67 (1.20, 2.33)	1.61 (1.15, 2.25)
*P* for trend	0.001	0.001	0.003
NHR			
Continuous	1.05 (1.01, 1.10)	1.05 (1.01, 1.10)	1.03 (0.98, 1.08)
Categories			
Q1	Reference	Reference	Reference
Q2	1.09 (0.79, 1.50)	1.09 (0.79, 1.50)	1.04 (0.75, 1.45)
Q3	1.44 (1.00, 2.07)	1.44 (1.00, 2.07)	1.36 (0.94, 1.96)
Q4	1.50 (1.05, 2.13)	1.50 (1.06, 2.12)	1.33 (0.93, 1.91)
*P* for trend	0.015	0.013	0.066
LHR			
Continuous	0.97 (0.85, 1.09)	0.97 (0.85, 1.09)	0.92 (0.80, 1.06)
Categories			
Q1	Reference	Reference	Reference
Q2	1.26 (0.93, 1.71)	1.25 (0.92, 1.69)	1.23 (0.90, 1.67)
Q3	1.09 (0.78, 1.52)	1.09 (0.78, 1.52)	1.03 (0.73, 1.45)
Q4	1.00 (0.71, 1.42)	1.00 (0.71, 1.42)	0.89 (0.62, 1.28)
*P* for trend	0.820	0.824	0.391
PHR			
Continuous	1.00 (0.99, 1.00)	1.00 (0.99, 1.00)	1.00 (0.99, 1.00)
Categories			
Q1	Reference	Reference	Reference
Q2	0.97 (0.73, 1.29)	0.97 (0.73, 1.28)	0.96 (0.72, 1.27)
Q3	1.05 (0.76, 1.46)	1.06 (0.76, 1.47)	1.00 (0.72, 1.39)
Q4	1.33 (0.99, 1.79)	1.34 (1.00, 1.79)	1.22 (0.90, 1.67)
*P* for trend	0.083	0.075	0.245
MHR			
Continuous	1.34 (0.85, 2.12)	1.35 (0.86, 2.11)	1.13 (0.70, 1.82)
Categories			
Q1	Reference	Reference	Reference
Q2	1.10 (0.74, 1.64)	1.10 (0.74, 1.63)	1.07 (0.72, 1.60)
Q3	1.25 (0.90, 1.73)	1.25 (0.90, 1.73)	1.16 (0.83, 1.63)
Q4	1.24 (0.86, 1.79)	1.24 (0.86, 1.78)	1.12 (0.77, 1.63)
*P* for trend	0.175	0.165	0.476

Model 1: no covariates were adjusted.

Model 2: age, gender, and race were adjusted.

Model 3: age, gender, race, marital status, education level, income-to-poverty ratio, BMI, smoking, alcohol use, diabetes, cardiovascular disease, hypertension, hyperlipidemia, and cancer were adjusted.

*p* < 0.05 was deemed statistically significant.

OR^1^, Odd ratio. 95% CI^2^, 95% confidence interval.

NPAR, neutrophil-percentage-to-albumin ratio; NPAR/10, NPAR divided by 10; NLR, neutrophil-to-lymphocyte ratio; NHR, neutrophil-to-high-density lipoprotein cholesterol ratio; LHR, lymphocyte-to-high-density lipoprotein cholesterol ratio; PHR: platelet-to-highdensity lipoprotein cholesterol ratio; MHR, Monocyte-to-high-density lipoprotein cholesterol ratio.

**Table 3:** Threshold effect analysis of systemic inflammatory indicators on psoriasis using a two-stage linear regression model in Model 3.

Threshold effect analysis	Psoriasis OR^1^ (95%CI^2^) *P*-value
NPAR	
Linear effect	1.005 (1.012, 1.100) 0.013
Inflection point of NPAR (K)	16.386
< K slope	1.119 (1.062, 1.181) <0.001
> K slope	0.885 (0.785, 0.983) 0.032
Log-likelihood ratio test	<0.001
NPAR/10	
Linear effect	1.702 (1.123, 2.591) 0.013
Inflection point of NPAR/10 (K)	1.639
< K slope	3.073 (1.816, 5.265) <0.001
> K slope	0.293 (0.089, 0.843) 0.032
Log-likelihood ratio test	<0.001
NLR	
Linear effect	1.081 (0.998, 1.161) 0.043
Inflection point of NLR (K)	3.269
< K slope	1.415 (1.225, 1.635) <0.001
> K slope	0.711 (0.529, 0.906) 0.013
Log-likelihood ratio test	<0.001
NHR	
Linear effect	1.049 (1.001, 1.098) 0.042
Inflection point of NHR (K)	4.286
< K slope	1.164 (1.048, 1.295) 0.005
> K slope	0.976 (0.891, 1.059) 0.583
Log-likelihood ratio test	0.030

Adjusted for age, gender, race, marital status, education level, income-to-poverty ratio, BMI, smoking, alcohol use, diabetes, cardiovascular disease, hypertension, hyperlipidemia, and cancer.

*p* < 0.05 was deemed statistically significant.

OR^1^: Odd ratio. 95% CI^2^: 95% confidence interval.

NPAR, neutrophil-percentage-to-albumin ratio; NPAR/10, NPAR divided by 10; NLR, neutrophil-to-lymphocyte ratio; NHR, neutrophil-to-high-density lipoprotein cholesterol ratio.

### 3.3 Subgroup analysis

**Figure 3:**
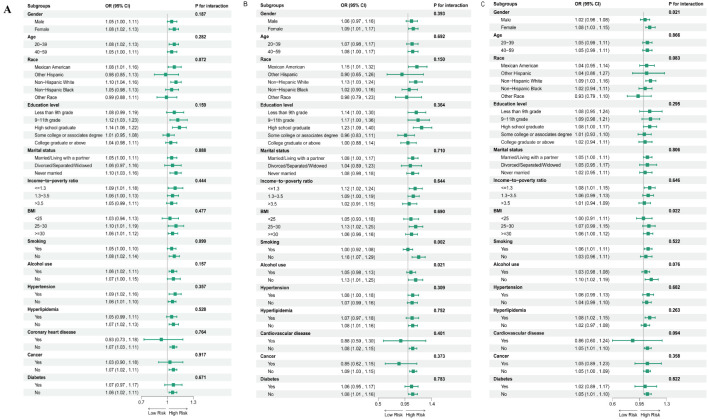
Subgroup analysis of assessing the association between quartile 4 of NPAR **(A)**, NLR **(B)** and NHR **(C)** with psoriasis (**Figure 3**), Adjustments were made for a range of demographic and health factors, including age, gender, race, marital status, education level, income-to-poverty ratio, BMI, smoking, alcohol usage, diabetes, cardiovascular disease, hypertension, hyperlipidemia, and cancer, with the stratification component in question being excluded. The figure depicts odds ratios (OR) represented by squares, and 95% CI indicated by horizontal lines.

Subgroup analyses were performed to assess whether specific characteristics such as age, gender, race, educational attainment, marital status, PIR, BMI, smoking habits, alcohol consumption, hypertension, hyperlipidemia, CVD, cancer history, and diabetes were associated with higher odds of psoriasis prevalence, and whether these associations held accurate across various demographic and clinical groups (**Figure 3**). Notably, no significant interactions were identified between these characteristics and the relationship between NPAR and higher odds of psoriasis prevalence. When examining the NHR quartiles, individuals in the highest quartile, particularly females (OR=1.08, 95% CI 1.03-1.15) and those with increased BMI (OR=1.06, 95% CI 1.00-1.12), were more likely to be diagnosed with psoriasis. Similarly, in the NLR cohort, individuals who smoked (OR=1.00, 95% CI 0.92-1.08) or consumed alcohol (OR=1.05, 95% CI 0.98-1.13) showed an elevated risk of psoriasis development.

### 3.4 ROC analysis

**Figure 4:**
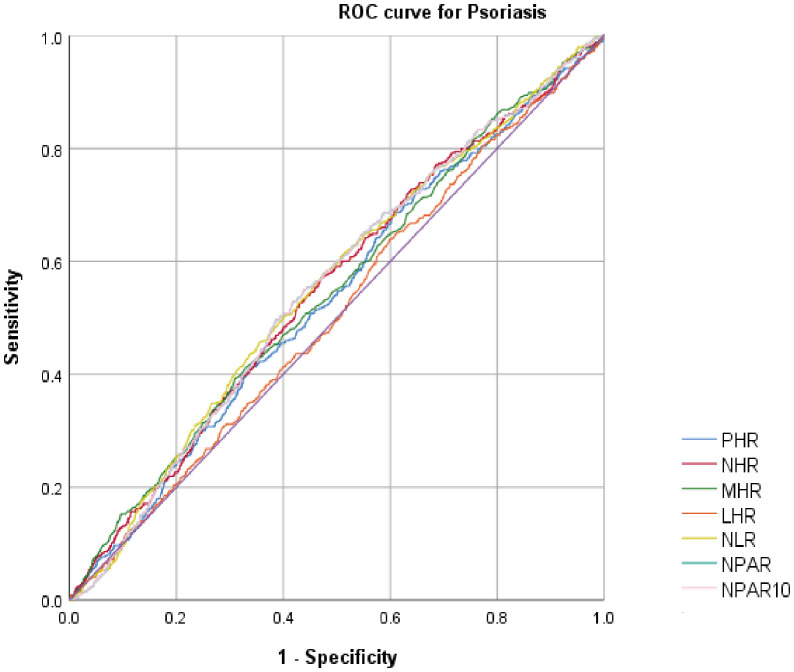
ROC curve for psoriasis diagnoses.

We calculated the AUC values to evaluate the predictive capacity of systemic inflammatory indicators (**Figure 4**). The results indicated that the AUC values for NHR, NLR, and NPAR were superior to those of the other inflammatory indicators. Additionally, **Table 4** shows that the AUC values for NPAR, NHR, and NLR were statistically significant (*P*<0.05).

**Table 4:** AUC values of systemic inflammatory indicators.

Test	AUC^1^	95%Cl^2^ low	95%Cl^2^ up	Best threshold	Specificity	Sensitivity	*P* for difference in AUC
**PHR**	0.537	0.510	0.564	45.137	0.001	1	0.010
**NHR**	0.553	0.526	0.580	0.600	0.002	0.998	<0.001
**MHR**	0.548	0.520	0.575	0.274	0.232	0.822	0.001
**LHR**	0.511	0.484	0.539	0.418	0.003	0.998	0.463
**NLR**	0.556	0.529	0.583	2.119	0.595	0.506	<0.001
**NPAR**	0.553	0.527	0.580	91.801	0.019	0.986	<0.001
**NPAR/10**	0.553	0.527	0.580	91.801	0.019	0.986	<0.001

*p* < 0.05 was deemed statistically significant.

AUC1, area beneath the curve.

95% Cl^2^, 95% confidence interval.

PHR, platelet-to-high-density lipoprotein cholesterol ratio; NHR, neutrophil-to-high-density lipoprotein cholesterol ratio; MHR, Monocyte-to-high-density lipoprotein cholesterol ratio; LHR, lymphocyte-to-high-density lipoprotein cholesterol ratio; NLR, neutrophil-to-lymphocyte ratio; NPAR, neutrophil-percentage-to-albumin ratio; NPAR/10, NPAR divided by 10.

## 4 Discussion

The current cross-sectional study revealed that NPAR, NLR, and NHR were significantly associated with the increased odds of psoriasis prevalence in the US population. However, LHR, PHR, and MHR showed no such association with psoriasis prevalence. In addition, upon adjusting for various covariates such as age, gender, race, marital status, education level, PIR, BMI, smoking, alcohol consumption, diabetes mellitus, CVD, hypertension, hyperlipidemia, and cancer, the association between NHR and the odds of psoriasis prevalence was no longer statistically significant. Prior research has suggested a non-linear relationship between NLR and both the prevalence and severity of psoriasis (14). In this study, we report for the first time a correlation between NPAR, NHR, and the incidence of psoriasis.

Our study has identified NPAR as a robust biomarker for systemic inflammation, with previous research highlighting its predictive capacity for various conditions, including acute kidney damage, cardiogenic shock, myocardial infarction, diabetic retinopathy, fatty liver disease (MASLD), depression, and cancer (15–17). Neutrophils, a critical component of human white blood cells are instrumental in orchestrating the inflammatory response. Albumin, on the other hand, exhibits anti-inflammatory and antioxidant properties that are modulated by inflammatory states, resulting in variations in concentration variations (18). In individuals with dietary deficits and inflammation, hypoalbuminemia frequently indicates a poor prognosis (11). Notably, our study reveals that NPAR is an exceptional predictor of psoriasis incidence, independent of covariates. The ease of measuring neutrophil percentage and albumin concentration render them valuable clinical indicators. NPAR outperforms other inflammatory blood biomarkers, such as the NLR and eosinophil-to-lymphocyte ratio (ELR), in predicting 5-year all-cause mortality, as evidenced by data from the NHANES database (19).

The NLR is indeed a valuable indicator for assessing systemic inflammation, offering a cost-effective and straightforward means to gauge the extent of inflammation, particularly in patients experiencing significant symptoms post-stress. It depends on data sets from conventional tests in laboratories and offers an affordable means to evaluate the severity of systemic inflammation, especially in patients who are very symptomatic after a stressful incident (7, 20). The NLR comprehensively reflects two distinct but complementary immune pathways: the innate immune response, represented by neutrophils, and the adaptive immune response, represented by lymphocytes. This ratio is not only a simple parameter to measure but also carries significant clinical implications. Research has linked NLR to pro-inflammatory cytokines, indicating its potential as an inflammation marker (21). Moreover, subgroup analyses have illuminated that, individuals within the highest NLR quartile (quartile 4), along with those who smoke or consume alcohol, face an elevated risk of developing psoriasis. These findings suggest that smoking and alcohol consumption are risk factors for the incidence of psoriasis.

Neutrophils play a pivotal role in chronic inflammatory and autoimmune diseases, and their involvement in psoriasis is well-documented by histopathological features like neutrophil-filled Munro microabscesses (22). In widespread pustular psoriasis, a characteristic neutrophil excess is evident (23). Previous studies have reported elevated NLR and platelet-to-lymphocyte ratios (PLR) in psoriasis patients (24), although these markers do not always reflect disease severity (25). Nonetheless, a decrease in NLR levels following psoriasis treatment highlights the significance of NLR levels in monitoring disease progression (26). NPAR, another marker of systemic inflammation, combines neutrophil counts with albumin levels (27). Albumin, a negative acute phase protein, is reduced in chronic inflammatory states (28), which may contribute to the elevated NPAR observed in psoriasis patients. Unlike lymphocyte counts, albumin levels are less affected by acute fluctuations and thus reflect long-term inflammation more consistently. This stability makes NPAR a potentially more accurate indicator of psoriasis disease activity compared to NLR (29).

High-density lipoprotein (HDL) has immunomodulatory, anti-inflammatory, anti-thrombotic, and antioxidant properties. These protective attributes have sparked interest in biomarkers related to HDL that may reflect underlying inflammatory processes. Notably, four novel markers have emerged: the lymphocyte-to-HDL cholesterol ratio (LHR), monocyte-to-HDL cholesterol ratio (MHR), neutrophil-to-HDL cholesterol ratio (NHR), and platelet-to-HDL cholesterol ratio (PHR). These ratios are calculated by contrasting HDL cholesterol levels with blood cell counts, shedding light on the association between lipid metabolism and inflammation (7). The NHR, especially, has garnered attention for its potential as a biomarker linking inflammation and lipid metabolism, providing insights into the interactions between these complex processes (19). Research has shown that the NHR has high predictive accuracy for a range of systemic conditions, including acute biliary pancreatitis, schizophrenia, bipolar disorder, hypertension, cardiovascular risk, and hepatocellular carcinoma (11). However, it’s worth noting that when confounding factors are considered, the association between the prevalence of psoriasis and NHR is insignificant.

Our findings highlight the potential role of NPAR and NLR as biomarkers for psoriasis and its associated comorbidities, including cardiovascular diseases (CVD) and metabolic syndrome. Systemic inflammation is a common link between psoriasis and these conditions (30, 31), with elevated levels of inflammatory markers like NLR and NPAR reflecting underlying inflammatory pathways. For example, NLR has been associated with increased cardiovascular risk and insulin resistance (32, 33), while NPAR, which combines neutrophil counts with albumin levels, may indicate long-term inflammatory states and metabolic dysregulation, such as hypoalbuminemia often observed in chronic diseases (34). These markers could help identify psoriasis patients at higher risk of developing CVD or metabolic syndrome, enabling earlier intervention and personalized treatment strategies. Future research should further explore the relationships between these markers and comorbid conditions, as well as their utility in clinical practice for risk stratification and therapeutic decision-making. There are benefits and drawbacks to our research. Firstly, its primary strength lies in the sufficient number of participants, which fortifies the credibility of the findings by aligning with previous study results. Secondly, the inclusion of a comprehensive set of covariates in the analysis ensures a higher degree of accuracy in the results. Finally, the present research is the first to investigate the association between psoriasis and NPAR, NHR, and other relevant inflammatory indicators. Nevertheless, the study still has limitations. The cross-sectional design of the study, allows for the exploration of associations but cannot establish causality. While our findings provide valuable insights into the relationships between systemic inflammatory markers (e.g., NPAR, NLR) and psoriasis, the temporal sequence of these associations remains unclear. Future prospective studies are needed to confirm these findings and explore potential causal mechanisms, particularly through longitudinal cohorts or interventional design. Another limitation is the small sample size in some subgroup analyses, which may have led to unstable estimates and reduced statistical power. For example, the association between NLR and psoriasis in certain subgroups (e.g., smokers) was based on limited data. Therefore, these results should be interpreted cautiously, and future studies with larger sample sizes are needed to validate these findings and explore subgroup differences more robustly. Furthermore, the reliance on self-reported data for psoriasis diagnosis may introduce bias, thereby compromising the accuracy of the results. Lastly, the absence of data on psoriasis severity hinders the statistical examination of its association with inflammatory indicators, reducing the comprehensiveness of your analysis.

## 5 Conclusion

NPAR and NLR have a strong positive correlation with the prevalence of psoriasis, while NHR does not show a statistically significant association. Furthermore, NPAR and NLR outperform other inflammatory indicators, such as PHR, NHR, MHR, LHR, in accuracy and discriminative ability. This indicates that these two indicators may be more useful in identifying and distinguishing psoriasis from other conditions.

## Data Availability

The datasets presented in this study can be found in online repositories. The names of the repository/repositories and accession number(s) can be found below: https://www.cdc.gov/nchs/nhanes/.
