# Correction: Association between systemic inflammation indicators and psoriasis: a cross-sectional study from NHANES

**DOI:** 10.3389/fimmu.2025.1690809

**Published:** 2025-11-06

**Authors:** Huizi Xiong, Zengyang Yu

**Affiliations:** 1 Department of Dermatology, The Fourth Affiliated Hospital of Soochow University, Suzhou Medical College, Soochow University, Suzhou, China; 2 Department of Dermatology, Shanghai Tenth People’s Hospital, Tongji University School of Medicine, Shanghai, China; 3 Institute of Psoriasis, Tongji University School of Medicine, Shanghai, China

**Keywords:** NHANES, psoriasis, NPAR, NHR, NLR

In the published article, there was an error in affiliation 1. Instead of “Department of Dermatology, The Fourth Affiliated Hospital of Soochow University/Suzhou DushuLake Hospital/Medical Center of Soochow University, Suzhou, Jiangsu, China”, it should be “Department of Dermatology, The Fourth Affiliated Hospital of Soochow University, Suzhou Medical College, Soochow University, Suzhou, China”.

In the published article, there was an error in the caption for **Figure 1**. The caption for the **Figure 1** was displayed as “Selection flowchart for this study”. The corrected caption appears below.

**Figure 1:**
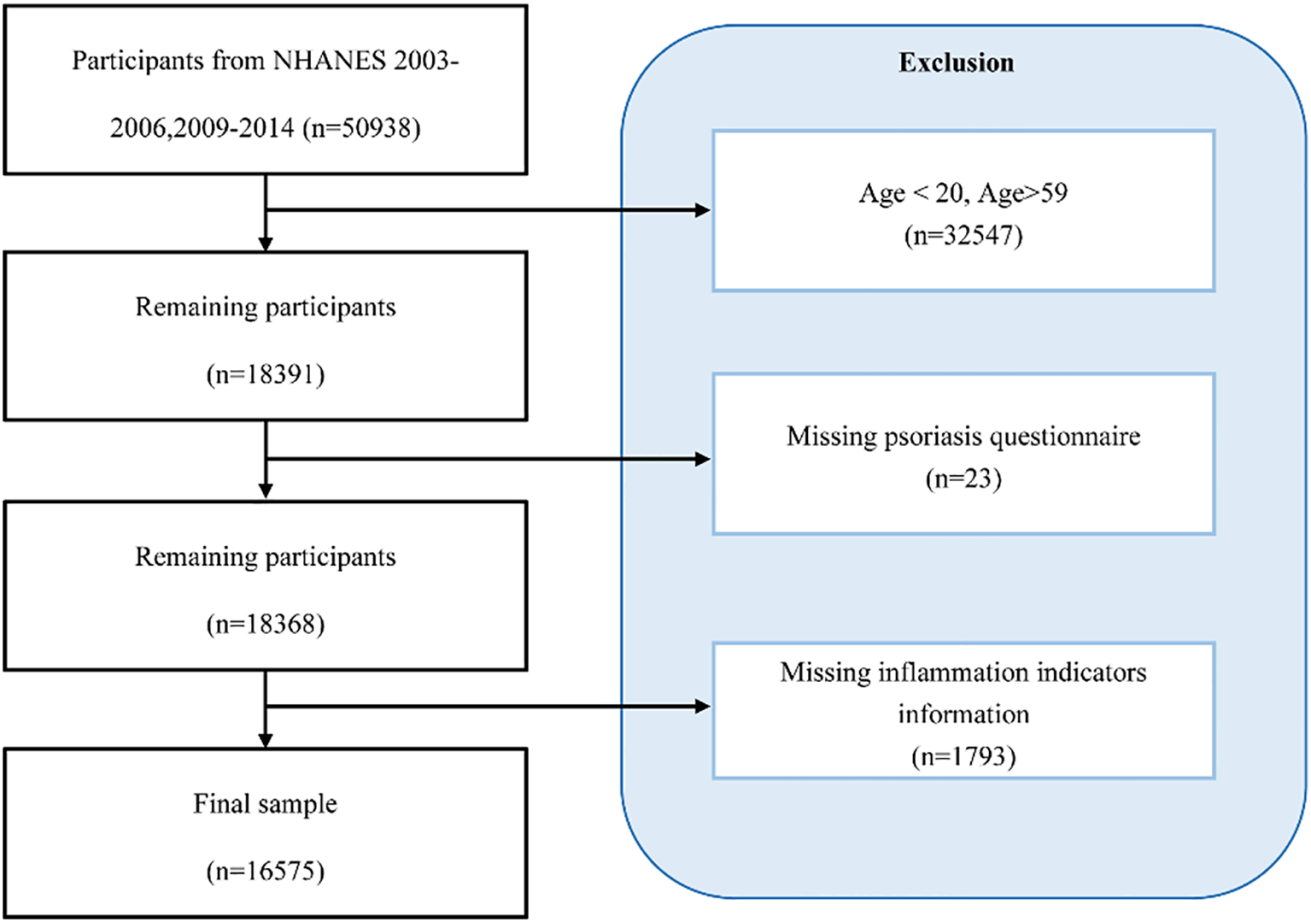
Participant selection flowchart for this study.

“Participant selection flowchart for this study.”

In the published article, the legend for **Table 1** was erroneously excluded. The corrected legend appears below.

**Table 1:** The baseline characteristics of participants enrolled in the NHANES cycles spanning from 2003–2006 and 2009-2014.

Variables	Overall	Psoriasis		*P*-value
	(n=16575)	NO (n=16143)	YES (n=432)	
**Gender, n (%)**				0.037
Male	7,945 (48%)	7,714 (48%)	231 (55%)	
Female	8,630 (52%)	8,429 (52%)	201 (45%)	
**Age, Mean ± SD**	38.76± (11.37)	38.75± (11.38)	39.20± (11.31)	0.495
**Age strata, n (%)**				0.134
20-39	8,626 (52%)	8,418 (52%)	208 (48%)	
40-59	7,949 (48%)	7,725 (48%)	224 (52%)	
**Race, n (%)**				0.613
Mexican American	2,911 (18%)	2,827 (18%)	84 (19%)	
Other Hispanic	1,283 (7.7%)	1,255 (7.8%)	28 (7.2%)	
Non-Hispanic White	7,127 (43%)	6,948 (43%)	179 (40%)	
Non-Hispanic Black	3,524 (21%)	3,429 (21%)	95 (20%)	
Other Race	1,730 (10%)	1,684 (10%)	46 (13%)	
**Education level, n (%)**				0.003
Less than 9th grade	1,294 (8.2%)	1,247 (8.1%)	47 (12%)	
9-11th grade	2,400 (15%)	2,339 (15%)	61 (13%)	
High school graduate	3,732 (23%)	3,650 (23%)	82 (17%)	
Some college or associate degree	5,237 (31%)	5,109 (31%)	128 (28%)	
College graduate or above	3,912 (23%)	3,798 (23%)	114 (30%)	
**Marital status, n (%)**				0.639
Married/Living with a partner	10,126 (61%)	9,855 (61%)	271 (63%)	
Divorced/Separated/Widowed	2,409 (15%)	2,339 (15%)	70 (15%)	
Never married	4,040 (25%)	3,949 (25%)	91 (22%)	
**Income-to-poverty ratio, n (%)**				0.070
≤1.3	5,327 (33%)	5,197 (33%)	130 (31%)	
1.3-3.5	6,086 (37%)	5,933 (37%)	153 (33%)	
>3.5	5,162 (30%)	5,013 (29%)	149 (35%)	
**BMI, n (%)**				0.502
<25	5,197 (35%)	5,079 (35%)	118 (32%)	
25-30	5,342 (34%)	5,199 (34%)	143 (34%)	
≥30	6,036 (31%)	5,865 (31%)	171 (34%)	
**Smoking, n (%)**				<0.001
Yes	7,086 (44%)	6,860 (44%)	226 (54%)	
No	9,489 (56%)	9,283 (56%)	206 (46%)	
**Alcohol use, n (%)**				0.984
Yes	12,351 (79%)	12,030 (79%)	321 (79%)	
No	4,224 (21%)	4,113 (21%)	111 (21%)	
**Hypertension, n (%)**				0.003
Yes	3,683 (22%)	3,550 (22%)	133 (30%)	
No	12,892 (78%)	12,593 (78%)	299 (70%)	
**Hyperlipidemia, n (%)**				<0.001
Yes	4,670 (30%)	4,505 (29%)	165 (40%)	
No	11,905 (70%)	11,638 (71%)	267 (60%)	
**Cardiovascular disease, n (%)**				0.514
Yes	187 (1.1%)	176 (1.1%)	11 (1.4%)	
No	16,388 (99%)	15,967 (99%)	421 (99%)	
**Cancer, n (%)**				0.016
Yes	638 (5.0%)	607 (4.9%)	31 (8.3%)	
No	15,937 (95%)	15,536 (95%)	401 (92%)	
**Diabetes, n (%)**				0.594
Yes	1,075 (5.5%)	1,041 (5.5%)	34 (6.2%)	
No	15,500 (94%)	15,102 (94%)	398 (94%)	
**C**	7.32± (2.20)	7.33± (2.21)	7.11± (1.98)	0.144
**Platelet (10^3^ cells/μL)**	255.92± (65.20)	255.79± (65.13)	260.29± (67.21)	0.310
**Lymphocyte (10^3^ cells/μL)**	2.17± (0.81)	2.17± (0.82)	2.10± (0.66)	0.211
**Monocyte (10^3^ cells/μL)**	0.55± (0.18)	0.54± (0.18)	0.55± (0.16)	0.171
**Neutrophil (10^3^ cells/μL)**	4.37± (1.74)	4.36± (1.74)	4.52± (1.65)	0.059
**Neutrophils percent (%)**	58.52± (8.97)	58.48± (8.97)	59.80± (8.63)	0.004
**Albumin (g/L)**	42.98± (3.50)	42.99± (3.50)	42.75± (3.34)	0.147
**HDL -C (mmol/L)**	1.37± (0.41)	1.37± (0.41)	1.34± (0.41)	0.179
**NPAR (dL/g)**	13.73± (2.60)	13.72± (2.60)	14.11± (2.56)	0.003
**NLR (10^9^/mmol)**	2.16± (1.05)	2.16± (1.05)	2.29± (0.94)	<0.001
**NHR (10^9^/mmol)**	3.51± (1.86)	3.51± (1.86)	3.70± (1.77)	0.014
**LHR (10^9^/mmol)**	1.75± (0.93)	1.75± (0.94)	1.72± (0.82)	0.800
**PHR (10^9^/mmol)**	202.85± (79.16)	202.65± (79.20)	209.33± (77.83)	0.063
**MHR (10^9^/mmol)**	0.44± (0.22)	0.44± (0.22)	0.45± (0.20)	0.098

*p* < 0.05 was deemed statistically significant; NHANES, National Health and Nutrition Examination Survey.

BMI, body mass index; HDL-C, high-density lipoprotein cholesterol; NPAR, neutrophil-percentage-to-albumin ratio; NLR, neutrophil-to-lymphocyte ratio; NHR, neutrophil-to-high-density lipoprotein cholesterol ratio; LHR, lymphocyte-to-high-density lipoprotein cholesterol ratio; PHR, platelet-to-high-density lipoprotein cholesterol ratio; MHR, Monocyte-to-high-density lipoprotein cholesterol ratio.

“*p < 0.05 was deemed statistically significant; NHANES: National Health and Nutrition Examination Survey; Abbreviations: BMI: body mass index; HDL-C: high-density lipoprotein cholesterol; NPAR: neutrophil-percentage-to-albumin ratio; NLR: neutrophil-to-lymphocyte ratio; NHR: neutrophil-to-high-density lipoprotein cholesterol ratio; LHR: lymphocyte-to-high-density lipoprotein cholesterol ratio; PHR: platelet-to-high-density lipoprotein cholesterol ratio; MHR: Monocyte-to-high-density lipoprotein cholesterol ratio.*”

In the published article, there was an error in the caption for **Table 2**. The caption for **Table 2** was displayed as “Associations between systemic inflammatory indicators and psoriasis in NHANES 2003-2006, 2009–2014 used multivariable logistic regression models”. The corrected caption appears below.

“Associations between systemic inflammatory indicators and psoriasis: Multivariable logistic regression analysis of NHANES 2003–2006 and 2009-2014.“

In the published article, there was an error in **Table 1**. There were mistakes in the description of the “HDL” and “Mean+ SD”, the correct descriptions were “HDL-C” and “Mean ± SD”. The corrected **Table 1** and its caption “The baseline characteristics of participants enrolled in the NHANES cycles spanning from 2003–2006 and 2009-2014” appears below.

In the published article, there was an error in Table 2. The column headings “Model 1 OR (95% CI)” “Model 2 OR (95% CI)” and “Model 3 OR (95% CI)” should be written instead as “Model 1 OR^1^ (95% CI^2^)” “Model 2 OR1 (95% CI^2^)” and “Model 3 OR^1^ (95% CI^2^)” respectively. The corrected Table 2 appears below.

In the published article, there was an error in **Table 3**. There was a mistake in the description of inflection point of “NPAR/100K”, the correct description is inflection point of “NPAR/10K”. Furthermore, the second column heading “Psoriasis OR (95%CI) *P*-value” should be corrected to read “Psoriasis OR^1^ (95%CI^2^) *P*-value”. The corrected **Table 3** and its caption “Threshold effect analysis of systemic inflammatory indicators on psoriasis using a two-stage linear regression model in Model 3” appears below.

In the published article, there was an error in Table 4. The fourth column heading “95%Cl up” should be corrected to “95%Cl^2^ up”. The corrected Table 4 appears below.

In the published article, there was an error in **Figure 4** as published. There was a mistake in the description of “NPAR10”, the correct description is “NPAR/10”. The corrected **Figure 4** and its caption “ROC curve for psoriasis diagnoses” appears below.

**Figure 4:**
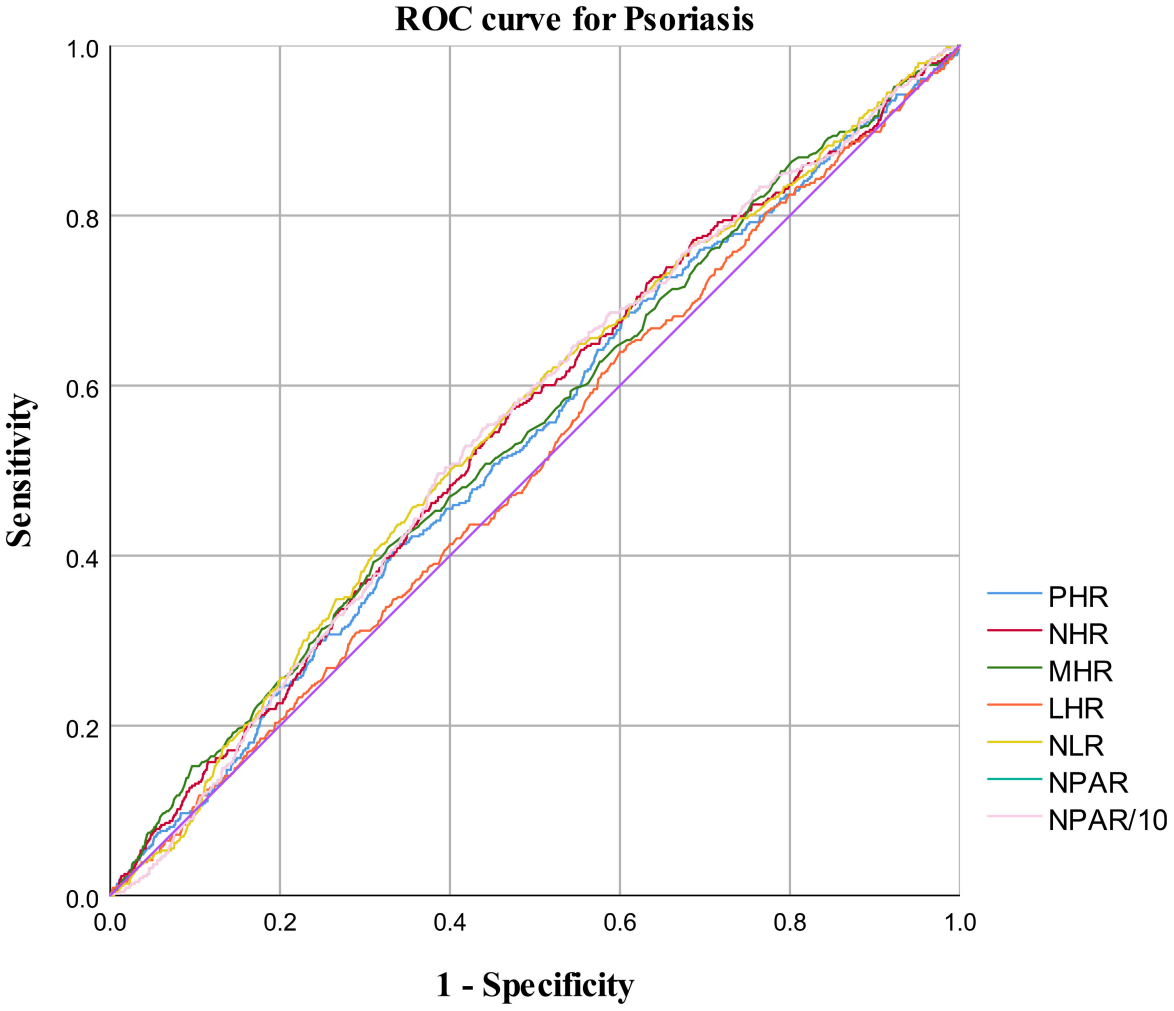
ROC curve for psoriasis diagnoses.

Reference “Zhao J, Zheng Q, Ying Y, Luo S, Liu N, Wang L, et al. Association between high-density lipoprotein-related inflammation index and periodontitis: Insights from nhanes 2009-2014. *Lipids Health Dis* (2024), 23(1): 321.doi:10.1186/s12944-024-02312-9” was not cited in the article. The citation has now been inserted in the section **Introduction**, paragraph 3 and should read:

“Similarly, other ratios such as NHR (neutrophil-to-hemoglobin ratio), LHR (lymphocyte-to-hemoglobin ratio), MHR (monocyte-to-hemoglobin ratio), and PHR (platelet-to-hemoglobin ratio) have also been identified as potential markers of inflammatory factors that play a critical role in many diseases (10, 11).”

Reference “Zhang Y, Qian H, Kuang Y H, Wang Y, Chen W Q, Zhu W, et al. Evaluation of the inflammatory parameters as potential biomarkers of systemic inflammation extent and the disease severity in psoriasis patients. Arch Dermatol Res.2024, 316(6): 229.doi:10.1007/s00403-024-02972-8.” was not cited in the article. The citation has now been inserted in the section Introduction, paragraph 4 and should read:

“The recent findings that psoriasis patients have higher levels of CRP (C-reactive protein), MHR (monocyte-to-hemoglobin ratio), NLR (neutrophil-to-lymphocyte ratio), and MLR (monocyte-to-lymphocyte ratio), and that these levels are positively correlated with the severity of the psoriasis condition, are significant (12).”

A correction has been made to **Abstract**, *Methods*, This sentence previously stated:

“We analyzed data from 16,575 adult participants in the National Health and Nutrition Examination Survey (NHANES) conducted between the years 2003–2004 and 2009-2014. We employed multivariable logistic regression and nonlinear curve fitting methods, which allowed us to evaluate the associations between psoriasis and systemic inflammation indicators such as NPAR, NLR, NHR, LHR, PHR (platelet-to-high-density lipoprotein cholesterol ratio), and MHR”

The corrected sentence appears below:

“We analyzed data from 16,575 adults in the National Health and Nutrition Examination Survey (NHANES) from 2003–2006 and 2009–2014. Six inflammatory ratios—neutrophil percentage-to-albumin ratio (NPAR), neutrophil-to-lymphocyte ratio (NLR), neutrophil-to-high-density lipoprotein cholesterol ratio (NHR), lymphocyte-to-high-density lipoprotein cholesterol ratio (LHR), platelet-to-high-density lipoprotein cholesterol ratio (PHR), and monocyte-to-high-density lipoprotein cholesterol ratio (MHR”

A correction has been made to **Introduction**, Paragraph 3. This sentence previously stated:

“The NPAR (neutrophil-to-albumin ratio), NHR (neutrophil-to-hemoglobin ratio), LHR (lymphocyte-to-hemoglobin ratio), MHR (monocyte-to-hemoglobin ratio), and PHR (platelet-to-hemoglobin ratio)”

The corrected sentence appears below:

“The neutrophil-percentage-to-albumin ratio (NPAR), the neutrophil-to-high-density lipoprotein cholesterol ratio (NHR), lymphocyte-to-high-density lipoprotein cholesterol ratio (LHR), monocyte-to-high-density lipoprotein cholesterol ratio (MHR), and platelet-to-high-density lipoprotein cholesterol ratio (PHR)”

In the published article, there was a lack of HDL-C description.

A correction has been made to **Introduction**, Paragraph 3. The additional sentence to be added to the end of the paragraph appears below:

“High-density lipoprotein cholesterol (HDL-C) is known for its anti-inflammatory properties. However, both its levels and function are compromised during inflammatory states. Furthermore, HDL-C engages in complex regulatory interactions with various immune cell types”

A correction has been made to **Methods**, *2.1 Population under investigation*. This sentence previously stated:

“A total of 16,424 individuals participated in both the “complete blood count, high-density lipoprotein cholesterol, albumin” examination and the “Psoriasis” questionnaire.”

The corrected sentence appears below:

“A total of 16,575 individuals participated in both the “complete blood count, high-density lipoprotein cholesterol, albumin” examination and the “Psoriasis” questionnaire.”

A correction has been made to **Methods**, *2.2 Measurement of systemic inflammation indicators NPAR, NHR, LHR, PHR and MHR*, Paragraph 1.

This sentence previously stated:

“The Beckman Coulter DxH 900 Automated Hematology Analyzer (Beckman Coulter, Brea, CA, USA) and NHANES derived CBC curves are used for hematological measurements, including hemoglobin levels, hemoglobin level, erythrocyte indices, and red and white blood cells” and “NPAR: (Percent neutrophils of total leukocyte count [%]) × 100/Albumin (g/CL);NHR: Neutrophil counts (10^9/L)/HDL (mmol/L); LHR: Lymphocyte counts (10^9/L)/HDL (mmol/L); PHR: Platelet counts (10^9/L)/HDL (mmol/L); MHR: Monocyte counts (10^9/L)/HDL (mg/dL)”

The corrected sentence appears below:

“The relevant laboratory measures were obtained from the NHANES database. HDL-C levels were assessed using an automatic device, with venous blood samples collected after an 8-hour fast. The standard reference range for HDL-C is 1.3–1.5 mmol/L for females and 1.0–1.5 mmol/L for males.” and “NPAR: (Percent neutrophils of total leukocyte count [%]) × 100/Albumin (g/dL);NHR: Neutrophil counts (10^9/L)/HDL (mmol/L);LHR: Lymphocyte counts (10^9/L)/HDL (mmol/L); PHR: Platelet counts (10^9/L)/HDL (mmol/L); MHR: Monocyte counts (10^9/L)/HDL (mmol/L); NLR: Neutrophil counts (10^9/L)/Lymphocyte counts (10^9/L)”

A correction has been made to **Methods**, *2.4 Covariates*. This sentence previously stated:

“Hyperlipidemia was defined using National Cholesterol Education Program criteria, incorporating LDL cholesterol levels ≥130 mg/dL, HDL cholesterol levels ≤40 mg/dL for males and ≤50 mg/dL for females, triglycerides levels ≥150 mg/dL, cholesterol levels ≥200 mg/dL, and the use of cholesterol-lowering medications.”

The corrected sentence appears below:

“Hyperlipidemia was defined using National Cholesterol Education Program criteria, incorporating LDL cholesterol ≥ 4.1 mmol/L, HDL-C ≤ 1.0 mmol/L in men or ≤ 1.3 mmol/L in women, triglycerides ≥ 2.3 mmol/L, total cholesterol ≥ 6.2 mmol/L, or current use of cholesterol-lowering medication.”

In the published article, there was an error in the legends for **Tables 2**–**4** as published. The following information was excluded from the legend of **Tables 2**–**4**: “OR^1^: Odd ratio. 95% CI^2^: 95% confidence interval”

The corrected **Tables 2**–**4**, and their legends, appear below.

**Table 2:** Associations between systemic inflammatory indicators and psoriasis: multivariable logistic regression analysis of NHANES 2003–2006 and 2009-2014.

	Model 1 OR^1^ (95% CI^2^)	Model 2 OR^1^ (95% CI^2^)	Model 3 OR^1^ (95% CI^2^)
NPAR			
Continuous	1.06 (1.01, 1.11)	1.06 (1.01, 1.11)	1.07 (1.01, 1.13)
Categories			
Q1	Reference	Reference	Reference
Q2	0.99 (0.69, 1.42)	1.00 (0.70, 1.43)	0.99 (0.69, 1.41)
Q3	1.26 (0.94, 1.69)	1.26 (0.94, 1.70)	1.25 (0.92, 1.70)
Q4	1.53 (1.09, 2.14)	1.54 (1.11, 2.15)	1.56 (1.11, 2.19)
*P* for trend	0.005	0.004	0.006
NPAR/10			
Continuous	1.73 (1.10, 2.72)	1.75 (1.12, 2.74)	1.90 (1.11, 3.26)
Categories			
Q1	Reference	Reference	Reference
Q2	0.99 (0.69, 1.42)	1.00 (0.70, 1.43)	0.99 (0.69, 1.41)
Q3	1.26 (0.94, 1.69)	1.26 (0.94, 1.70)	1.25 (0.92, 1.70)
Q4	1.53 (1.09, 2.14)	1.54 (1.11, 2.15)	1.56 (1.11, 2.19)
*P* for trend	0.005	0.004	0.006
NLR			
Continuous	1.11 (1.03, 1.19)	1.11 (1.04, 1.20)	1.10 (1.02, 1.18)
Categories			
Q1	Reference	Reference	Reference
Q2	0.98 (0.68, 1.42)	0.99 (0.69, 1.43)	0.98 (0.68, 1.41)
Q3	1.24 (0.88, 1.73)	1.24 (0.88, 1.74)	1.22 (0.87, 1.71)
Q4	1.66 (1.19, 2.32)	1.67 (1.20, 2.33)	1.61 (1.15, 2.25)
*P* for trend	0.001	0.001	0.003
NHR			
Continuous	1.05 (1.01, 1.10)	1.05 (1.01, 1.10)	1.03 (0.98, 1.08)
Categories			
Q1	Reference	Reference	Reference
Q2	1.09 (0.79, 1.50)	1.09 (0.79, 1.50)	1.04 (0.75, 1.45)
Q3	1.44 (1.00, 2.07)	1.44 (1.00, 2.07)	1.36 (0.94, 1.96)
Q4	1.50 (1.05, 2.13)	1.50 (1.06, 2.12)	1.33 (0.93, 1.91)
*P* for trend	0.015	0.013	0.066
LHR			
Continuous	0.97 (0.85, 1.09)	0.97 (0.85, 1.09)	0.92 (0.80, 1.06)
Categories			
Q1	Reference	Reference	Reference
Q2	1.26 (0.93, 1.71)	1.25 (0.92, 1.69)	1.23 (0.90, 1.67)
Q3	1.09 (0.78, 1.52)	1.09 (0.78, 1.52)	1.03 (0.73, 1.45)
Q4	1.00 (0.71, 1.42)	1.00 (0.71, 1.42)	0.89 (0.62, 1.28)
*P* for trend	0.820	0.824	0.391
PHR			
Continuous	1.00 (0.99, 1.00)	1.00 (0.99, 1.00)	1.00 (0.99, 1.00)
Categories			
Q1	Reference	Reference	Reference
Q2	0.97 (0.73, 1.29)	0.97 (0.73, 1.28)	0.96 (0.72, 1.27)
Q3	1.05 (0.76, 1.46)	1.06 (0.76, 1.47)	1.00 (0.72, 1.39)
Q4	1.33 (0.99, 1.79)	1.34 (1.00, 1.79)	1.22 (0.90, 1.67)
*P* for trend	0.083	0.075	0.245
MHR			
Continuous	1.34 (0.85, 2.12)	1.35 (0.86, 2.11)	1.13 (0.70, 1.82)
Categories			
Q1	Reference	Reference	Reference
Q2	1.10 (0.74, 1.64)	1.10 (0.74, 1.63)	1.07 (0.72, 1.60)
Q3	1.25 (0.90, 1.73)	1.25 (0.90, 1.73)	1.16 (0.83, 1.63)
Q4	1.24 (0.86, 1.79)	1.24 (0.86, 1.78)	1.12 (0.77, 1.63)
*P* for trend	0.175	0.165	0.476

Model 1: No covariates were adjusted.

Model 2: Age, gender, and race were adjusted.

Model 3: Age, gender, race, marital status, education level, income-to-poverty ratio, BMI, smoking, alcohol use, diabetes, cardiovascular disease, hypertension, hyperlipidemia, and cancer were adjusted.

*p* < 0.05 was deemed statistically significant.

OR^1^, Odd ratio. 95% CI^2^, 95% conﬁdence interval.

NPAR, neutrophil-percentage-to-albumin ratio; NPAR/10, NPAR divided by 10; NLR, neutrophil-to-lymphocyte ratio; NHR, neutrophil-to-high-density lipoprotein cholesterol ratio; LHR, lymphocyte-to-high-density lipoprotein cholesterol ratio; PHR: platelet-to-high-density lipoprotein cholesterol ratio; MHR, Monocyte-to-high-density lipoprotein cholesterol ratio.

**Table 3:** Threshold effect analysis of systemic inflammatory indicators on psoriasis using a two-stage linear regression model in Model 3.

Threshold effect analysis	Psoriasis OR^1^ (95%CI^2^) *P*-value
NPAR	
Linear effect	1.005 (1.012, 1.100) 0.013
Inflection points of NPAR (K)	16.386
< K slope	1.119 (1.062, 1.181) <0.001
> K slope	0.885 (0.785, 0.983) 0.032
Log-likelihood ratio test	<0.001
NPAR/10	
Linear effect	1.702 (1.123, 2.591) 0.013
Inflection points of NPAR/10 (K)	1.639
< K slope	3.073 (1.816, 5.265) <0.001
> K slope	0.293 (0.089, 0.843) 0.032
Log-likelihood ratio test	<0.001
NLR	
Linear effect	1.081 (0.998, 1.161) 0.043
Inflection points of NLR (K)	3.269
< K slope	1.415 (1.225, 1.635) <0.001
> K slope	0.711 (0.529, 0.906) 0.013
Log-likelihood ratio test	<0.001
NHR	
Linear effect	1.049 (1.001, 1.098) 0.042
Inflection points of NHR (K)	4.286
< K slope	1.164 (1.048, 1.295) 0.005
> K slope	0.976 (0.891, 1.059) 0.583
Log-likelihood ratio test	0.030

Adjusted for age, gender, race, marital status, education level, income-to-poverty ratio, BMI, smoking, alcohol use, diabetes, cardiovascular disease, hypertension, hyperlipidemia, and cancer.

*p* < 0.05 was deemed statistically significant.

OR^1^: Odd ratio. 95% CI^2^: 95% confidence interval.

NPAR, neutrophil-percentage-to-albumin ratio; NPAR/10, NPAR divided by 10; NLR, neutrophil-to-lymphocyte ratio; NHR, neutrophil-to-high-density lipoprotein cholesterol ratio.

**Table 4:** AUC values of systemic inflammatory indicators.

Test	AUC^1^	95%Cl^2^ low	95%Cl^2^ up	Best threshold	Specificity	Sensitivity	*P* for difference in AUC
PHR	0.537	0.510	0.564	45.137	0.001	1	0.010
NHR	0.553	0.526	0.580	0.600	0.002	0.998	<0.001
MHR	0.548	0.520	0.575	0.274	0.232	0.822	0.001
LHR	0.511	0.484	0.539	0.418	0.003	0.998	0.463
NLR	0.556	0.529	0.583	2.119	0.595	0.506	<0.001
NPAR	0.553	0.527	0.580	91.801	0.019	0.986	<0.001
NPAR/10	0.553	0.527	0.580	91.801	0.019	0.986	<0.001

*p* < 0.05 was deemed statistically significant.

AUC^1^, area beneath the curve.

95% Cl^2^, 95% confidence interval.

PHR, platelet-to-high-density lipoprotein cholesterol ratio; NHR, neutrophil-to-high-density lipoprotein cholesterol ratio; MHR, Monocyte-to-high-density lipoprotein cholesterol ratio; LHR, lymphocyte-to-high-density lipoprotein cholesterol ratio; NLR, neutrophil-to-lymphocyte ratio; NPAR, neutrophil-percentage-to-albumin ratio; NPAR/10, NPAR divided by 10.

